# L2 Proficiency Pairing, Task Type and L1 Use: A Mixed-Methods Study on Optimal Pairing in Dyadic Task-Based Peer Interaction

**DOI:** 10.3389/fpsyg.2021.699774

**Published:** 2021-06-17

**Authors:** Lili Tian, Yan Jiang

**Affiliations:** School of Foreign Languages, Renmin University of China, Beijing, China

**Keywords:** Chinese EFL learners, L2 proficiency pairing, task type, L1 use, negotiation of meaning, peer interaction

## Abstract

While task-based peer interaction in dyads has been commonly practiced in English as a foreign language (EFL) classrooms, how to pair learners in dyadic tasks has always been a concern of teachers and researchers. This study examined learner proficiency pairing, task type and L1 use by Chinese EFL learners in two dyadic speaking tasks. Thirty-six participants were paired according to their oral English proficiency levels into: same-level pairs (high-high; medium-medium; low-low), and mixed-level pairs (high-low). All pairs completed two types of speaking tasks—information-gap and opinion-exchange. Quantitative results showed a significant difference between low-low pairs and other pairs in the amount of L1 use. Low-level learners produced significantly more L1 words and turns when paired with other low-level peers (low-low) than with high-level peers (high-low) in both types of tasks. Qualitative analysis further indicated that the mixed-level (high-low) pairs produced more opportunities for negotiation of meaning than the same-level (low-low) pairs during the interactional episodes where the L1 served various functions. The study offers pedagogical implications for EFL teachers about how to optimally pair learners to maximize their language development.

## Introduction

Second language (L2) interactionists have long claimed that conversational interaction between non-native speakers through communicative tasks can enhance speaking opportunities (Long and Porter, 1985, Varonis and Gass, 1985) and facilitate negotiation of meaning (Long, 1983; Pica and Doughty, 1985). Negotiation of meaning has been deemed a vital way for learners to resolve communication difficulties during learner-learner interaction (Long, 1983), which might improve comprehensible input (Krashen, 1985) and facilitate opportunities for output (Swain, 1985), thus effectively promoting L2 development (Pica and Doughty, 1985; see a review in Loewen and Sato, 2018).

One pedagogical issue during such learner-learner task-based oral interaction was that learners did not always stay in the target language if they shared the same first language (L1; Littlewood, 2007; Carless, 2008; Wolthuis et al., 2019). Learners' overuse of L1 might reduce their opportunities for L2 production, thus hindering L2 development (Krashen, 1985; Brooks and Donato, 1994; Hird, 1996). Previous studies investigating L1 use during task-based peer interaction mainly examined the amount and functions of L1 use, with the assumption that modest use of L1 would result in the least loss of opportunities for L2 production (Storch and Aldosari, 2010; Azkarai and García Mayo, 2015). However, it remains under-explored whether this assumption holds true. In addition to the amount and functions of L1 use, research findings on L1 use and L2 production should also be examined from the perspective of opportunities for negotiation of meaning (Long and Porter, 1985). Compared with the same functions served by L2, whether these L1 functions, served by the modest use of L1, affected opportunities for negotiation of meaning through interactional moves during the pertinent interactional episodes still puzzles classroom practitioners.

In order to optimize learners' language use and promote L2 learning when implementing learner-learner activities in class, researchers have also investigated the factors impacting the amount of L1 use and its functions, such as learner L2 proficiency (Swain and Lapkin, 2000; De la Colina and Garcia Mayo, 2009; Storch and Aldosari, 2010, 2012; Kim, 2020), task type (Azkarai and García Mayo, 2015), task repetition (Azkarai and García Mayo, 2017; Payant and Reagan, 2018), interactional patterns (Storch and Aldosari, 2012; Xu and Kou, 2017), and instructional settings (Lin, 2015; García Mayo and Ángeles Hidalgo, 2017; Vázquez and Ordóñez, 2019). These studies investigating the interface between L2 proficiency and L1 use in different dyadic oral tasks have reached the consensus that low-level learners are likely to produce more L1 than higher-level learners in different dyadic tasks (Frawley and Lantolf, 1985; Swain and Lapkin, 2000; Storch and Wigglesworth, 2003; De la Colina and Garcia Mayo, 2007; DiCamilla and Anton, 2012). However, it remains under-researched as to whether learners of same- and mixed-levels of L2 proficiency would trigger different L1 use in different dyadic oral tasks. The few studies that have examined proficiency pairing and L1 use (Storch and Aldosari, 2010; Kou and Li, 2019) measured learners' proficiency levels based on a combination of previous ratings rather than one rigorous test, and administered different tasks without adopting a counter-balanced task design to eliminate task sequence effect.

With the prevalence of communicative language teaching (Nunan, 2003) and task-based language teaching (Ellis, 2017) since the 1990s, task-based peer interaction has been widely practiced in large classes in East Asian English as a foreign language (EFL) contexts (Littlewood, 2007; Li, 2014) in order to offer learners more opportunities to use L2 (Philp et al., 2014). However, research on L1 use and its impacting factors contextualized in East Asian EFL classrooms with large class sizes is still scarce (Kou and Li, 2019), especially in oral English classrooms where the task objective is to improve oral English proficiency.

This study aimed to examine the effects of L2 proficiency pairing and task type on learners' L1 use by employing a rigorous research design and delving into the opportunities for negotiation of meaning through interactional moves involving L1 use in a Chinese EFL context. We explored the extent to which EFL learners employ their L1 and the functions L1 use serves, and if any differences could be observed between same- and mixed-level pairs in different speaking tasks. We also looked into the opportunities for negotiation of meaning through interactional moves where the major L1 functions occurred. Understanding the nature of L1 use by dyads of mixed proficiency pairings in different tasks would help EFL teachers make pedagogical decisions on how to pair learners for task-based oral interactions.

## Literature Review

### Task Type and L1 Use in Dyadic Task-Based Oral Interaction

Research on naturalistic codeswitching has reached a consensus on the role of L1 as an indicator of bilingual competence rather than a defect (Myers-Scotton and Jake, 1995; Li, 2000). However, a protracted debate about L1 use in L2 classrooms took place between L2 exclusivists (Krashen, 1985) and L1 optimalists (Cook, 2001; Macaro, 2005; De Guerrero, 2018). For L2 exclusivists, learners' L1 use during oral interaction detracted from L2 use, thereby lessening opportunities for L2 acquisition. However, L1 optimalists argued that learners' judicious use of L1 could be a facilitator rather than a hindrance.

Optimalists gained support from a sociocultural perspective (Frawley and Lantolf, 1985; Vygotsky, 1986). Scaffolding could occur not only between a more capable peer/expert and a less competent partner/novice (Ohta, 1995) but also between novices through dialogic interaction (Storch, 2002). During collaborative task-based social activities L1 use could reduce anxiety and enhance the affective environment for learning (Auerbach, 1993; Neokleous, 2016), and promote verbal interaction and task completion via metatalk and orientational talk (Brooks and Donato, 1994). L1 use could also help establish intersubjectivity and externalize inner speech (Anton and DiCamilla, 1998), focus attention and highlight discrepancies through private speech (DiCamilla and Anton, 2004; Jiménez Jiménez, 2015), and serve as a mediating scaffolding strategy to maintain self-regulation in the dialogue (Villamil and De Guerrero, 1996; De Guerrero, 2018). A recent quasi-experimental study by Zhang (2019) empirically consolidated the role of L1 use in facilitating the understanding of lexico-grammatical aspects in the writing of collaborative texts.

Since the end of twentieth century, a number of empirical studies started to research learners' L1 use in task-based oral interaction in L2 classrooms. For example, Hird (1996) was among the earliest to examine the amount and functions of L1 use by a small group of four Chinese middle school students. He found that about one third of the participants' talk was in L1 Chinese. L1 served such functions as signaling direct quotations, specifying a particular addressee, reiterating for emphasis, and distinguishing between objectivity and subjectivity.

Studies in the early twenty-first century started to focus on learners' L1 use in relation to the oral interaction for writing tasks. For example, Swain and Lapkin (2000) examined the L1 turns and functions used by 22 pairs with a higher proficiency level completing two tasks (dictogloss and jigsaw) in French immersion contexts. The written stories of learners were rated in terms of both content and language, and students were labeled as high-achieving and low-achieving students based on their performance. No significant difference in L1 use was found due to the small sample size and high degree of variability, but high-achieving pairs generally produced fewer L1 turns, and the dictogloss task elicited more L1 turns than the jigsaw task. Low-achieving students had a stronger need to use L1, and tended to use more L1 for the jigsaw task. L1 was used to move the task along, focus attention on vocabulary and grammar, and achieve interpersonal interaction. Storch and Wigglesworth (2003) also found great variability between different pairs and tasks in an ESL setting. Two pairs who shared Chinese as their L1 used L1 more extensively (between 25 and 50%).

Despite the variation in the amount of L1 used by the participants in the abovementioned studies, they all acknowledged the facilitative role of L1 in task-based oral interaction. However, these studies only examined task type effects on L1 use, without looking at the effects of learners' L2 proficiency pairings on their L1 use.

### L2 Proficiency Pairing, Task Type, and L1 Use in Dyadic Task-Based Oral Interaction

Toward the end of the first decade of the 2000s, researchers began to focus on the effects of same-level proficiency pairings on learners' L1 use in relation to task types. De la Colina and Garcia Mayo (2007, 2009) examined elementary level, low-low (L-L) proficiency Spanish EFL learner pairs. Participants performing dictogloss (75%) and text reconstruction (78%) tasks generated more L1 words than they did during the jigsaw task (55%). They used L1 most frequently for metacognitive talk, especially in the text reconstruction task (De la Colina and Garcia Mayo, 2009). DiCamilla and Anton (2012) examined both L-L (low-low) and H-H (high-high) pairs in a collaborative writing task. However, they did not test students' proficiency level before pairings, with Spanish-major seniors deemed as high-level students and freshmen as low-level ones in an American university. L-L pairs (70–80%) used far more L1 words than H-H pairs (0–3%). H-H pairs tended to only use L1 to define tasks, whereas L-L pairs used L1 for an array of functions, from solving lexical problems to supporting the interpersonal relationship.

Different from the above studies, Azkarai and García Mayo (2015, 2017) explored speaking and writing task type effects on L1 use by same-level proficiency EFL learners. In the first study, two speaking tasks (picture placement and picture difference) with full attention to meaning were compared with two combined speaking and writing tasks (dictogloss and text editing) focusing on formal linguistic aspects (Azkarai and García Mayo, 2015). They found that combined speaking and writing tasks generated more L1 use than speaking tasks only. L1 was used for 15.41% of turns. Minor L1 use was for metacognitive and off-task purposes and predominant L1 turns were used for vocabulary searches and phatics. The later study examined the impact of task repetition on L1 use and reported a significant decrease in the amount of L1 use with task repetition (Azkarai and García Mayo, 2017).

To the best of our knowledge, only a limited number of studies have examined the L1 use of both same- and mixed-level pairs in task-based oral interaction. Storch and Aldosari (2010) paired 30 EFL first-year undergraduates into H-H, L-L, and H-L pairs based on a combination of L2 ratings. The participants completed three tasks (jigsaw, composition, and text-editing) in 3 weeks. Overall, the pairs used L1 for a total of 7% of words and 16% of turns, and there were more predominant L1 turns (61%) than minor L1 turns. L-L pairs only produced more L1 words on the text-editing task (29%), compared with that of H-H (9%) and H-L pairs (11%). Overall, H-L and L-L pairs (19%) produced more L1 turns than H-H pairs (11%). The H-L pairs used L1 most frequently for task management, while the L-L pairs used L1 for a wider range of functions. A dominant-passive relationship existed only in the H-L pairs, where the high-level peer acted as the dominant one, authoritatively directing task completion while the low-level peer was relatively passive. In contrast, the low-level learners in L-L pairs exhibited a collaborative relationship, in that they supported each other whenever a need for assistance arose.

Extending the previous research on the effects of dyadic interaction patterns and proficiency pairings on L2 language-related production (Watanabe and Swain, 2007; Kim and McDonough, 2008; Dao and McDonough, 2017). Storch and Aldosari (2012) examined the role of proficiency pairings and dyadic relationships on the amount of L2 use and language-related production in a collaborative writing task. They found that L2 proficiency pairings greatly impacted language-related production, but did not impact frequency of L2 words and turns as much. The H-H pairs produced on average 7% L1, whereas the other pairs (H-L, L-L) only 3%. They pointed out that “optimal pairing” depended on the goal of the activity, and recommended L-L pairing when the goal was to develop fluency, because L-L pairs could produce longer L2 turns (p. 47). It should be noted that Storch and Aldosari (2010, 2012) all focused on the oral interaction during collaborative writing tasks.

In a Chinese EFL university context, Kou and Li (2019) examined the effects of L2 proficiency pairing and speaking task type on L1 use. They found a modest use of L1 words (3.7%) and turns (17.7%). A significant difference was found in L1 words and turns between the same- and mixed-level pairings. L-L pairs produced significantly more L1 words and turns than H-H pairs, and no significant difference was found between L-L and H-L pairs. Task type exerted no significant effects. There was also no significant difference between L2 proficiency pairings in terms of functions served by L1 use.

Despite the non-significant difference in L1 use between same- and mixed-level pairs, the above studies reported mixed findings in terms of the amount of L1 words and turns used. Additionally, they measured their learners' proficiency levels based on a combination of previous ratings rather than one rigorous test, and administered their tasks without adopting a counter-balanced task design to eliminate task sequence effect. Most of previous studies examined the oral interaction during collaborative writing tasks rather than speaking tasks.

Notably, these above findings on L1 use and functions could hardly give a full picture of its impact on opportunities for L2 production. Previous studies on the efficacy of learner-learner interaction measured opportunities for L2 production by opportunities for negotiation of meaning through interactional moves (Pica et al., 1996; Iwashita, 2001; Gilabert et al., 2009). Long (1996) proposed three interactional moves—clarification request, confirmation check, and comprehension check—which might lead to opportunities for negotiation of meaning (Iwashita, 2001; Loewen and Sato, 2018). The efficacy of learner-learner interaction was measured by opportunities for negotiation of meaning through interactional moves and was affected by a range of learner and task factors, such as learner L2 proficiency (Pica et al., 1996; Iwashita, 2001; Watanabe and Swain, 2007) and task type (Doughty and Pica, 1986; Iwashita, 2001; Gilabert et al., 2009). For example, one-way tasks elicited more modifications of output than two-way tasks (Iwashita, 2001). Information gap tasks were shown to result in more negotiation of meaning than opinion gap tasks (Gilabert et al., 2009). High-high (H-H) proficiency dyads were found to perform meaning negotiation differently from low-low (L-L) proficiency dyads (Pica et al., 1996). Mixed-level dyads produced more interactional moves and opportunities for negotiation of meaning than same-level dyads (Porter, 1986; Yule and Macdonald, 1990; Iwashita, 2001), and resolved language-related issues more successfully (Kim and McDonough, 2008). There is a strong need to look at whether L1 use in these studies affected opportunities for negotiation of meaning during the pertinent interactional episodes.

This study thus aimed to fill these gaps by investigating the role of same- and mixed-level L2 proficiency pairings in both the amount of L1 use and the functions it served, and whether these L1 functions affected opportunities for negotiation of meaning in the pertinent interactional episodes. This study followed a rigorous research design, by: (1) focusing on two types of speaking tasks, which were common practices in EFL university classrooms with large class sizes; (2) adopting a counter-balanced task design so as to eliminate the potential impact of task sequence on task performance; (3) administering an oral English proficiency test to accurately measure learners' L2 proficiency levels before pairing; (4) diversifying the L2 proficiency pairings used in previous studies by incorporating medium-medium (M-M) learners. In addition to the type and frequency of L1 functions, this study also delved into whether these L1 functions affected opportunities for negotiation of meaning through interactional moves in those episodes where L1 functions occurred, so as to gain a full picture of the functions served by L1 use. The following research questions were then examined:

1. Do different proficiency pairings and tasks affect the amount of L1 words and turns used by Chinese EFL learners in dyadic task-based oral interactions?
2. Do different proficiency pairings and tasks affect the type of functions the L1 use serves, and the interactional moves elicited by these functions?

## Materials and Methods

### Context and Participants

The study was conducted in a top-tier university in Beijing, China, where English is a compulsory subject for first-year students. These students have learned English for about 9 to 12 years. They were non-English major students, majoring in varieties of subjects in social science (see Table 1). They received roughly 3 h of English courses per week: 1.5 h each for English Listening and Speaking, and English Reading and Writing courses.

**Table 1:** Participants' gender and subject background information in different language proficiency pairings.

**L2 proficiency pairing**	**Participants' gender and subject background**				
L-L (3 pairs)	Deng-Wan (M-F) Finance- Chinese literature	Xiong-Wang (M-F) Economics- Economics	Li-Pan (F-F) Human resources- Human resources	Dropouts	
M-M (5 pairs)	Liu-Rui (F-F) Economics- Economics	Hui-Chou (F-F) Law-Law	Gong-Gao (F-F) International relations	Cui-Zhao (F-F) Business- Finance	Hou-He (F-F) Law- Business
H-H (5 pairs)	Shi-Zhou (F-F) Business- Business	Zhu-Xi (M-F) Business- Finance	Qili-Fan (F-F) Journalism- Science	Xu-Handi (M-F) Business- Finance	Wei-Sun (M-F) Business- Statistics
H-L (5 pairs)	Huang-Ting (F-F) International relations- International relations	Shi-Sun (M-M) Science- Information	Tang-Zihui (F-F) Journalism- Information	Xu-Qian (F-F) Business- Journalism	Sui-Shan (M-F) International relations

*H, high; M, medium; L, low; M, male; F, female*.

An invitation to participate in our study was sent to students in the three English Listening and Speaking classes taught by the first author, and fifty-two students initially agreed to participate. An oral proficiency interview was then administered to these students to measure their English oral proficiency level. Forty students were finally selected as the target participants due to the study's proficiency pairing needs. They were then paired into four different types of proficiency pairing based on their English oral proficiency levels: same-level pairs (H-H; M-M; L-L), and mixed-level pairs (H-L), with five pairs for each pairing type. However, due to two L-L pairs dropping out in the second round of data collection, only the interaction data of the 36 students who attended both data collection sessions were analyzed. These 36 students, with an average age of 19, were randomly paired, disregarding gender and subject backgrounds (see Table 1).

### Oral Proficiency Interview

An interview was conducted by the two authors to rate the participants' oral English proficiency levels. It included two parts: self-introduction and a paired oral task. The rubric of the interview followed the five scales of International English Language Testing System (IELTS) proficiency guidelines: fluency and coherence, pronunciation, grammatical range and accuracy, lexical resources, and discourse management. The two interviewers scored each student separately and an agreement on the proficiency level of the participants was then reached with an inter-rater reliability of 0.94. For the convenience of pairing, participants' English oral proficiency levels were labeled roughly as low, medium, or high.

### Tasks

This study examined two types of speaking task: an information-gap task and an opinion-exchange task (see Table 2). According to Nakahama et al. (2001), informational tasks are more structured two-way information-gap tasks, where learners are asked to exchange information they have in advance with their peers. On the other hand, conversational tasks are less structured and more open-ended opinion exchange tasks where learners are asked to exchange their own viewpoints on a given topic with their peers.

**Table 2:** The list of speaking tasks.

**Session**	**Topic**	**Speaking tasks**	**Task type**
Week 1	English mania	Exchange information on English mania between two professors (Prof. David Johnson and Prof. Chen Ping)	Information-gap
		Exchange your viewpoints on the effects of English mania	Opinion-exchange
Week 2	How to live longer	Exchange your viewpoints on the listed advices of how to live longer	Opinion-exchange
		Exchange information on body-building programs between a university student and a consultant from HOSA fitness club	Information-gap

Different from the collaborative writing tasks used in previous studies (Storch and Aldosari, 2010, 2012), the two task types examined in this study were speaking tasks only with no written component. These two task types were used for the following reasons: (1) our participants were quite familiar with them; (2) both task types were meaning-focused (i.e., learners concentrate on meaning rather than grammatical features, which are not the teaching focus of university-level English); and (3) these two task types differ in cognitive complexity (opinion-exchange tasks are more demanding because they elicit learners' personal viewpoints with supporting reasons).

### Data Collection

The data was collected in the autumn term of the academic year 2013–2014. Thirty-six participants completed four tasks in two data collection sessions, with two tasks in each week. The participants were allowed 8 min to complete each task. The task sequence was counter-balanced by administering the information-gap task first followed by the opinion-exchange task in the first week, with a reversed sequence in the second week. The two rounds of task completion helped achieve the counter-balanced task design, eliminating any potential task sequence effects on participants' task performance. The oral interaction of all dyads was audio-recorded, with one audio-recorder in front of each pair. There were roughly 850 min of audio recordings and 40,339 total words in the transcript.

### Data Analysis

#### L1 Words and Turns

Previous studies have employed different units of analysis to measure the amount of learners' L1 use, either only the number of L1 turns (Swain and Lapkin, 2000; Azkarai and García Mayo, 2015), or only the number of L1 words (De la Colina and Garcia Mayo, 2009), or both (Dörnyei et al., 2000; Storch and Aldosari, 2010). To gain a comprehensive insight into participants' L1 use in oral interactions, both the number of L1 turns and L1 words were examined in the present study. Following the categorization of L1 turn types in previous studies (Swain and Lapkin, 2000; Storch and Aldosari, 2010; Azkarai and García Mayo, 2015), L1 turns were categorized into predominant and minor turns according to the proportion of L1 and L2 words in each turn.

The audio recordings of participants' oral interactions were first transcribed verbatim. The amount of Chinese characters and English words was then counted for each participant and in each task, together with the amount and frequency of L1 turns. The quantitative data on the percentage of language use was entered into SPSS for statistical analysis. Descriptive statistics were first extracted to gain their mean and standard deviation. Inferential analysis was then implemented for between-group and within-group differences. The Kolmogorov-Smirnov test was used to examine the normality of data distribution before executing parametric (ANOVA) or non-parametric tests (Kruskal-Wallis). Total words were normally distributed (D[36] = 0.12, *p* > 0.05), whereas percentages of L1 words (D[36] = 0.29, *p* < 0.001) and L1 turns (D[36] = 0.24, *p* < 0.001) were not. The two authors conducted all the L1 counting and coding, and agreement was reached for any inconsistencies.

#### Codification of L1 Functions

We integrated the L1 function categories of Storch and Aldosari (2010) and Azkarai and García Mayo (2015), and formed a working list of L1 functions (see Table 3). The two authors separately and meticulously examined the working list of L1 functions with the data, and the frequency of L1 functions was then counted accordingly. We reached a 96% agreement on the codification, and all the discrepancies were resolved upon discussion. Grammar-related talk was excluded from the list and the subsequent function-related analysis, because no students produced grammar-related talk of L1 during their oral interaction in this study.

**Table 3:** A working list of L1 functions.

**Categories**	**Definitions**	**Examples**
Metacognitive talk	L1 was used to talk about the task, such as planning, organizing, monitoring, setting goals, and checking comprehension Azkarai and García Mayo, 2015, p. 557	Pan: *Because we don't have the chance. As the Chinese environment we all speak Chinese with each other. 总结一下我们的(summarize our opinion), 就刚开始先说一下(just say it at the beginning), first we should deliver the beginning. Hello, everyone, I am talking about blabla*
Vocabulary	L1 was used in deliberations over word/sentence meaning, word searches, and word choice Storch and Aldosari, 2010, p. 361; Azkarai and García Mayo, 2015, p. 58	Sun: …… *Only the towns, the people in the town can understand them. They just abandon the language and learn the 普通话(mandarin Chinese)*
Grammar	L1 was used to discuss issues related to grammar Storch and Aldosari, 2010, p. 361; Azkarai and García Mayo, 2015, p. 557	*Excluded*
Mechanics	L1 was used to discuss punctuation, spelling, and pronunciation Storch and Aldosari, 2010, p. 361	Deng: *And also decrease anxious 这怎么读(how to pronounce this)*
Phatics	L1 expressions were used to establish social contact and to express sociability rather than specific meaning Azkarai and García Mayo, 2015, p. 558	Handi: *I live in XXX university, Zhixing No. 2* Xu: *202 吧(a modal particle), 就(just like this). May I know something about your hobbies*
Off-task	L1 was used as casual talk, unrelated to the task Azkarai and García Mayo, 2015, p. 557	Xu: *Ok. Schedules, every Tuesday, Thursday, and Saturday* Handi: 为什么要戴帽子*(Why do you wear a hat)* Xu: 因为我昨天晚上没洗头*(because I didn't wash my hair last night)* Handi: 啊交代了*(Ah! You confessed)* 这个理由好*(This is a good reason)* Xu: *Every Tuesday, Thursday, and Saturday*

According to the three interactional moves proposed by Long (1996), we examined the interactional episodes where the same functions of L1 and L2 occurred as incidences of opportunities for negotiation of meaning. The pertinent interactional episodes where these L1 functions occurred were contrasted with the same episodes served by L2 in terms of the interactional moves and the opportunities for negotiation of meaning elicited by these moves. Two frequently used functions of L1 were selected as examples.

## Results

### Amount of L1 Use

#### L1 Words

Table 4 shows the number of total words and the percentage of L1 words in relation to different proficiency pairings. Predictably, the L-L pairs produced the least total words, and the H-H pairs the most. The percentage of L1 words used by all the pairs was fairly low, at 2.9 percent. M-M pairs produced the least L1 words during their oral interaction (0.7%), and L-L pairs used the most (10%). The H-L pairs produced only 1.5% L1 words. The low-level students in the L-L and H-L pairs seemed to be producing L1 words in different ways. On average, the low-level students in L-L pairs produced far more L1 words (10%) than those in H-L pairs (1.5%).

**Table 4:** The number of total words and percentage of L1 words per proficiency pairing.

	**Total words (L1+L2)**			**L1 words**			**L1 words percentage**		
	***N***	**Minimum**	**Maximum**	**N**	**Minimum**	**Maximum**	**Percent**	**Minimum**	**Maximum**
L-L (3 pairs)	6,132	801	1,199	613.4	17.18	236.9	10.0	1.6	22.8
M-M (5 pairs)	10,363	529	1,394	68.4	0	48	0.7	0	3.4
H-H (5 pairs)	12,055	461	1,840	292.4	0	146	2.4	0	12.7
H-L (5 pairs)	11,379	536	1,685	171.9	0	43	1.5	0	4.9
Total (18 pairs)	39,929	461	1,840	1146.1	0	236.9	2.9	0	22.8

The Kruskal-Wallis test showed a significant difference between proficiency pairings for both the information-gap (*X*^2^ = 14.7, *df* = 3, *p* = 0.002) and opinion-exchange tasks (*X*^2^ = 12.05, *df* = 3, *p* = 0.007). Wilcoxon's signed ranks test revealed no significant difference between the L1 percentages of these two task types (*Z* = −1.8, *p* = 0.072) for all proficiency pairings (see Table 5).

**Table 5:** The percentage of L1 words per task type and proficiency pairing.

	**Information-gap task**		**Opinion-exchange task**	
	**Mean**	***SD***	**Mean**	***SD***
L-L (3 pairs)	8.02	9.4	11.4	8.5
M-M (5 pairs)	0.3	0.6	1.0	1.9
H-H (5 pairs)	2.2	4.8	2.0	3.7
H-L (5 pairs)	1.1	1.2	2.1	2.98
Total (18 pairs)	2.3	5.1	3.3	5.6

To summarize, low-level learners produced significantly more L1 words when paired with other low-level peers than with high-level peers in both types of task. There were no task type effects on L1 use.

#### L1 Turns

Table 6 presents the frequency and type of L1 turns in relation to proficiency pairings. The percentage of total L1 turns was fairly low, at 6%. M-M and H-L produced more total turns, whereas L-L produced the least. Predictably, L-L produced the most L1 turns (17%), much more than the other pairs. In terms of L1 turn types, there were more minor L1 turns (55%) than predominant ones (45%). While H-H pairs produced more predominant L1 turns, the other pairs produced many more minor L1 turns than predominant L1 turns.

**Table 6:** The frequency and type of L1 turns per proficiency pairing.

	**Total turns (L1+L2)**	**L1 turns**		**L1 turns: types**			
				**Predominant L1 turns**		**Minor L1 turns**	
	**N**	**N**	**Percentage of L1 turns**	**N**	**Percentage of L1 turns**	**N**	**Percentage of L1 turns**
L-L (3 pairs)	551	92	17	41	45	51	55
M-M (5 pairs)	943	18	2	4	22	14	78
H-H (5 pairs)	791	56	7	33	59	23	41
H-L (5 pairs)	839	37	4	13	35	24	65
Total (18 pairs)	3124	203	6	91	45	112	55

Table 7 shows the frequency of L1 turns in relation to task type and proficiency pairing. The Kruskal-Wallis test showed a significant difference in L1 turn percentage between proficiency pairings for both information-gap (*X*^2^ = 13.1, *df* = 3, *p* = 0.004) and opinion-exchange tasks (*X*^2^ = 10.2, *df* = 3, *p* = 0.017). The low-level students in the L-L and H-L pairs seemed to produce L1 turns in different ways. On average, the low-level students in L-L pairs produced far more L1 turns than those in H-L pairs in both tasks.

**Table 7:** The frequency of L1 turn percentage per task type and proficiency pairing.

	**Information-gap task**		**Opinion-exchange task**	
	**Mean (%)**	**SD (%)**	**Mean (%)**	**SD (%)**
L-L (3 pairs)	15.8	15.0	21.2	14.9
M-M (5 pairs)	1.0	2.3	3.1	5.2
H-H (5 pairs)	5.8	10.5	5.0	5.9
H-L (5 pairs)	3.2	2.9	5.7	7.3
Total (18 pairs)	5.4	9.5	7.4	10.1

### Functions Served by L1

#### The Frequency of L1 Functions

Table 8 presents the frequency and percentage of the functions L1 use served in relation to proficiency pairing. In total, the main function of L1 use was vocabulary talk (54%), followed by metacognitive talk (23%). In terms of different proficiency pairings, L-L pairs mostly used L1 for vocabulary (51%) and metacognitive talk (42%), whereas H-H pairs mostly used it for off-task (50%) and vocabulary (35%) talk. Interestingly, H-L pairs mostly used L1 for vocabulary (89%) rather than metacognitive talk (2%). Table 9 presents the similar frequency distribution of L1 functions for the two tasks, with vocabulary as the most used L1 function, followed by metacognitive talk.

**Table 8:** The frequency and percentage of L1 functions per proficiency pairing.

	**Metacognitive talk**		**Vocabulary**		**Mechanics**		**Phatics**		**Off-task**		**Total**
	***N***	**%**	***N***	**%**	***N***	**%**	***N***	**%**	***N***	**%**	***N***
L-L (3 pairs)	46	42	56	51	1	1	7	6	0	0	110
M-M (5 pairs)	0	0	3	60	0	0	2	40	0	0	5
H-H (5 pairs)	4	6	23	35	0	0	6	9	33	50	66
H-L (5 pairs)	1	2	39	89	0	0	4	9	0	0	44
Total (18 pairs)	51	23	121	54	1	0.4	19	8	33	14.6	225

**Table 9:** The frequency and percentage of L1 functions per task type.

	**Metacognitive talk**		**Vocabulary**		**Mechanics**		**Phatics**		**Off-task**		**Total**
	***N***	**%**	***N***	**%**	***N***	**%**	***N***	**%**	***N***	**%**	***N***
Information-gap task	22	21	56	53	1	1	10	9	17	16	106
Opinion-exchange task	29	24	65	55	0	0	9	8	16	13	119

The abovementioned quantitative findings indicated a significant difference in the amount of L1 words and turns between H-L and L-L pairs. The L1 functions of these two types of pairings were mainly differentiated in relation to metacognitive and vocabulary talk. We examined the interactional moves in the task-related interactional episodes where L1 and L2 use served the same functions (metacognitive and vocabulary talk), with regard to the opportunities for negotiation of meaning which might be elicited by these moves.

#### The Interactional Moves of Metacognitive Talk by H-L and L-L Pairs

We examined the interactional moves in the task-related interactional episodes where L1 and L2 use served the same metacognitive talk functions, with regard to the opportunities for negotiation of meaning which might be elicited by these moves. The following examples (1) and (2), produced by L-L (Pan-Rui) and H-L (Huang-Ting) pairs during the same opinion-exchange task on the effects of English mania, are good cases in point.

Example (1)

1. Pan (L): Hello, everyone, today I'm talking about the mania of English.
2. Rui (L): So what's your view?
3. Pan: (*I should say it by myself*), (*this is not a dialogue*), (*I say it by myself*). I hold a positive view about the phenomena, because I think English helps …… Another reason is that English allows …

Example (2)

1. Huang (H): I hold a positive view about the English mania. How about you?
2. Ting (L): I hold a positive view. So our team holds a positive view?
3. Huang: It is too early to make a conclusion. So what is your reason?
4. Ting: The reason?
5. Huang: A better future we can have and a better job for high salary. Our ability to speak English, you can see here, because many well-pay jobs require knowledge of English.
6. Ting: Yeah.
7. Huang: And learning of English will surely have a positive effect on any of us who study English as a second language. This is the first reason. That is to say, better life and better job.
8. Ting: I think the second reason is … (pauses here for 3 seconds) English can help …err (pauses here for 2 s) help us to learn foreign country, and people and culture.

The episodes above revealed different interactional moves and opportunities for negotiation of meaning. In the L-L pair (Example 1), Pan initiated the conversation in turn 1, with the intention of stating her viewpoint and pretending that she was giving a presentation on her own in class, which was her understanding of the task. When Rui interrupted her by requesting clarification in turn 2, Pan became anxious in turn 3 and used L1 to inform him of her understanding of how the task should be completed. Pan used L1 as a strategy to immediately bring the conversation back on track in order to move the task along. However, this quick resolution of communication difficulty over task management effectively shut down Rui's opportunity to negotiate.

However, the H-L pair in Example (2) demonstrated completely different interactional features when a disagreement on completing the same task arose. In turn 2, Ting (L) requested clarification of their view, which was obviously too early to do so according to the teacher's task instructions. In turn 3, Huang (H) explained this to Ting and guided her to state her reasons. When Ting again requested clarification in turn 4, Huang set an example for her in turn 5. As Huang continued to elaborate more on her view in turn 7, Ting seemed inspired and voluntarily took the turn to join the conversation in turn 8, which could be deemed as successful pushed output. Although the same communication difficulty in task management was resolved more slowly in this example compared with Example (1), with the assistance of Huang, the high-level peer, further clarification requests generated more opportunities for both learners to negotiate meanings in L2.

It can be seen that, compared with L-L pairs who tended to use L1 as pedagogical strategies, the H-L pairs generated more interactional moves for metacognitive talk and opportunities for negotiation of meanings in L2.

#### The Interactional Moves of Vocabulary Talk by H-L and L-L Pairs

The same goes to the interactional moves of vocabulary talk. When completing an information-gap task about a university student consulting fitness club programs, the L-L pair in Example (3) and H-L pair in Example (4) encountered the same lexical difficulty of understanding the word *fatigue*.

Xiong in the L-L pair requested clarification in turn 2 to indicate a linguistic difficulty, and Wang repeated the L2 word and offered an L1 equivalent immediately afterwards in turn 3 before continuing talking. However, when receiving Shan's (L) clarification request in turn 2, Sui (H) in the H-L pair repeated the L2 phrase and offered an L2 explanation in turn 3. Shan immediately followed this with a confirmation check by repeating the L2 synonym with a rising intonation in turn 4, which was confirmed by affirmative feedback from Sui in turn 5. Again, although the same lexical difficulty was resolved more slowly than in Example (3), with the assistance of Sui, the high-level peer, the clarification request generated a confirmation check that allowed more opportunities for the low-level learner to produce the L2 synonym herself, thus extending her focus on and understanding of the meaning of the unknown word.

Example (3)

1. Wang (L): It can reduce body fat, keep fatigue away.
2. Xiong (L): keep what away?
3. Wang: fatigue, (*fatigue*), can help you manage stress …

Example (4)

1. Sui (H): And in the mental level, it can keep fatigue away.
2. Shan (L): keep what?
3. Sui: keep fatigue away, that means you will get less stressful and more relaxing.
4. Shan: maybe relaxing?
5. Sui: Right! And …

It can be seen from the above examples that L-L pairs used L1 in metacognitive and vocabulary strategies to quickly resolve communication problems during the interaction. However, with the aid of high-level peers, the H-L pairs produced more opportunities for negotiation of meaning through more interactional moves. This offered further evidence for the tendency that low-level students in L-L pairs produced more L1 words and turns, and fewer opportunities for negotiation of meaning, than those in the H-L pairs.

## Discussion

The present study investigated the role of L2 proficiency pairing and task type in the amount and functions of L1 used by Chinese EFL learners engaging in dyadic task-based oral interaction. The dyadic interactional negotiation of the pairs was also examined.

The participants in our study used L1 words in oral interaction to a fairly limited extent, at only 2.9%, lower than that in Storch and Aldosari (2010). Similarly, the total of L1 turns produced by our participants was also fairly low, at 6%, lower than Azkarai and García Mayo (2015), and much lower than Swain and Lapkin (2000). That is probably because our participants were from a prestigious Chinese university and had a relatively higher English proficiency.

In terms of L2 proficiency pairings, this study found a significant difference in the use of L1 words and turns between L-L pairs and all the other pairs in both tasks. This finding is inconsistent with those of Storch and Aldosari (2010, 2012) and Kou and Li (2019). The differences in L1 words and turns in these studies may be caused by the diverse L2 proficiency levels of their participants. Our participants had a relatively higher English proficiency. Despite the judicious use of L1 as communicative strategies (Macaro, 2005), English proficiency level is one of the important indicators of the percentage of L1 use (Azkarai and García Mayo, 2015). The task type may be another reason. Our study adopted speaking tasks commonly practiced in Chinese university EFL listening and speaking course contexts. Previous studies have mostly examined the oral interaction for completing writing tasks (Storch and Aldosari, 2010). Azkarai and García Mayo, 2015, who examined speaking and writing tasks, reported that combined speaking and writing tasks generated more L1 use than speaking tasks only.

In terms of task type effects, there was no significant difference in the amount of L1 words between different dyads. Our participants produced more minor L1 turns than predominant ones, particularly for opinion-exchange tasks. While H-H pairs produced more predominant L1 turns, the other pairs produced more minor L1 turns, with M-M and H-L pairs having produced far more minor L1 turns than predominant L1 turns. The analysis on L1 functions of H-H pairs showed that 50% of their L1 use, in predominant L1 turns, was off-task talk. These findings were similar to those of Azkarai and García Mayo, 2015, but were inconsistent with those of Storch and Aldosari (2010) who reported more predominant L1 turns than minor ones for all proficiency pairings. Despite the same first-year undergraduate EFL context for all these studies, the present study was conducted at a prestigious university in mainland China where the undergraduates possessed a higher proficiency in all subjects including English. Such an inconsistency would be further explained by the different functions L1 served for both studies.

Our findings contribute to the existing literature by revealing the impacts of different L2 proficiency pairings on the amount of L1 use. A significant difference was found in the amount of both L1 words and turns between L-L pairs and all the other pairs (H-H, M-M, H-L) in both tasks. This finding indicates that low-level learners, when paired with other low-level learners, produced significantly more L1 words and turns than when paired with high-level learners. Early learner-learner oral interaction studies informed us that L-L pairs might produce more negotiated meaning (Pica et al., 1996). While negotiating with the same low-level peers, learners felt no embarrassment (Varonis and Gass, 1985) and grasped any opportunities to use their shared mother tongue to enhance their interaction (Brooks and Donato, 1994).

The second research question queried the functions of L1 use under different proficiency pairings and task types. We found that L1 served a wide range of functions to facilitate interaction and task completion, lending support to the findings of previous studies (Storch and Aldosari, 2010; Azkarai and García Mayo, 2015). However, L1 use in this study was mainly for vocabulary talk, followed by metacognitive talk, which was inconsistent with previous studies (Swain and Lapkin, 2000; De la Colina and Garcia Mayo, 2009; Storch and Aldosari, 2010; Azkarai and García Mayo, 2015). This might again be related to the relatively higher language proficiency of our participants who focused more on meaning in the oral interactions.

Our findings could be explained from a sociocultural perspective (Vygotsky, 1978). The high-level peers in the H-L pairs acted as experts to deliberately provide scaffolding for the low-level peers for task management, while the low-level learners in the L-L pairs used L1 to help each other in managing and completing the tasks. Both high-level learners as experts and low-level learners as novices, and low-level learners as collaborative peers were able to offer scaffolded help to each other as social beings through oral interaction to achieve self-regulation and move across their zone of proximal development (Frawley and Lantolf, 1985; Brooks and Donato, 1994). High-level peers could use L2 as a mediating strategy to guide the low-level peer in H-L dyads toward task completion, while low-level peers in the L-L dyads used L1 as a semiotic mediator to achieve the same function. Peer scaffolding occurred between peers of the same and different proficiency levels through dyadic interaction (Storch, 2002).

In addition, Macaro et al. (2009) proposed a model of Teacher as Dictionary and Dictionary Designer. In this model, nonnative speaker teachers could have two languages (L1 and L2) at their disposal to facilitate learners' vocabulary acquisition. In the present study, the high-level peers in the H-L pairs acted in the role of non-native speaker teacher and provided their low-level peers with lexical support in L2, while low-level peers in the L-L pairs offered lexical support in L1. L1 or L2 vocabulary explanation functioned as a mediating strategy, helping learners tackle lexical problems and move the task along.

This study also delved into the interactional negotiation of L1 functions by contrasting them with the functions served by L2, and demonstrated the effects of different proficiency pairings on the opportunities for negotiation of meaning through interactional moves. Although L-L dyads quickly resolved communication difficulties by employing L1 in metacognitive and linguistic strategies, with the aid of high-level peers, the H-L dyads produced more opportunities for negotiation of meaning than the L-L dyads. This finding lent support to previous findings that mixed-level dyads produced more interactional moves and opportunities for modified output than same-level dyads (Porter, 1986; Yule and Macdonald, 1990; Iwashita, 2001). Mixed-level learner-learner interaction offered more opportunities for negotiation of meaning and modified output through interactional moves (Swain, 1985; Long, 1996), which in turn might enhance L2 development (Loewen and Sato, 2018).

We need to acknowledge the limitations of our study. Firstly, we are unsure how much language learning or gains low-level learners achieved through peer interaction. Secondly, we only reported those selected interactional moves in those interactional episodes involving two L1 functions, metacognitive and vocabulary talk. Future research could examine whether and how low-level learners could make progress through peer interaction, and also quantitatively explore the causal relationship of L1 use and opportunities for negotiation of meaning.

## Conclusion and Implications

This study examined the effects of different language proficiency pairs and task type on the L1 use of Chinese EFL learners in dyadic task-based oral interaction. We found a significant difference between L-L pairs and other pairs in both the amount and functions of L1 use in both speaking tasks. Low-level learners produced significantly more L1 when paired with the same low-level peers than with high-level peers. Although the amount of L1 words and turns used by the L-L dyads was modest, fewer opportunities for negotiation of meaning were detected in their interactional episodes.

The findings offer some pedagogical implications for EFL teachers about how to effectively implement dyadic task-based oral activities. Firstly, the two-way information-gap task and opinion-exchange role-play task were both suitable for EFL teachers to assign dyadic oral tasks without worrying about students generating too much L1 use. Secondly, EFL teachers could pair same-level learners of medium to high proficiency and mixed-level learners of high and low proficiency in L2 classrooms: this would not result in a significant increase in L1 use. It is better to pair low-level learners with high-level peers so as to produce more opportunities for negotiation of meaning and avoid generating significantly more L1 use, which might better facilitate learners' target language production and learning.

## Data Availability Statement

The raw data supporting the conclusions of this article will be made available by the authors, without undue reservation.

## Ethics Statement

The studies involving human participants were reviewed and approved by School of Foreign Languages, Renmin University of China. The participants provided their written informed consent to participate in this study.

## Author Contributions

LT conceived the initial idea, fine-tuned by YJ, designed the study, collected and analyzed the data, and drafted the manuscript. YJ collected and analyzed the data, revised, proofread, and finalized the manuscript for submission. All authors contributed to the manuscript and approved the submitted version.

## Conflict of Interest

The authors declare that the research was conducted in the absence of any commercial or financial relationships that could be construed as a potential conflict of interest.
